# Understanding liver regeneration to bring new insights to the mechanisms driving cholangiocarcinoma

**DOI:** 10.1038/s41536-017-0018-z

**Published:** 2017-05-22

**Authors:** R. V. Guest, L. Boulter, B. J. Dwyer, S. J. Forbes

**Affiliations:** 1 MRC Centre for Regenerative Medicine, University of Edinburgh, Edinburgh bioQuarter, 5 Little France Drive, , Edinburgh, EH16 4UU UK; 2 Institute for Genetics & Molecular Medicine, University of Edinburgh, Crewe Road, , Edinburgh, EH4 2XU UK

## Abstract

Cancer frequently arises in epithelial tissues subjected to repeated cycles of injury and repair. Improving our understanding of tissue regeneration is, therefore, likely to reveal novel processes with inherent potential for aberration that can lead to carcinoma. These highly conserved regenerative mechanisms are increasingly understood and in the liver are associated with special characteristics that underlie the organ’s legendary capacity for restoration of size and function following even severe or chronic injury. The nature of the injury can determine the cellular source of epithelial regeneration and the signalling mechanisms brought to play. These observations are shaping how we understand and experimentally investigate primary liver cancer, in particular cholangiocarcinoma; a highly invasive malignancy of the bile ducts, resistant to chemotherapy and whose pathogenesis has hitherto been poorly understood. Interestingly, signals that drive liver development become activated in the formation of cholangiocarcinoma, such as Notch and Wnt and may be potential future therapeutic targets. In this review, we summarise the work which has led to the current understanding of the cellular source of cholangiocarcinoma, how the tumour recruits, sustains and is educated by its supporting stromal environment, and the tumour-derived signals that drive the progression and invasion of the cancer. With few current treatments of any true efficacy, advances that will improve our understanding of the mechanisms driving this aggressive malignancy are welcome and may help drive therapeutic developments.

## Introduction

An enormous unmet clinical need exists for novel therapies in primary liver tumours; in particular cholangiocarcinoma (CC); a cancer of the bile ducts. This aggressive malignancy confers a notoriously poor prognosis; the current overall 5 year survival in the US is less than 17.5% (ref. 1). Patients often present too late for the only curative procedure- surgical resection, and investigations to obtain tissue for diagnostic confirmation are invasive and often inconclusive. There is currently no serum biomarker of the disease, which would aid early diagnosis. Carbohydrate antigen (CA) 19-9 is a circulating marker widely used for disease monitoring, however, its poor sensitivity and specificity, particularly in the context of cholangitis or cholestasis make it unsuitable for early disease detection.^2^ The bile ducts can be sometimes accessed endoscopically and cellular material obtained via brushing. Much effort has been made to improve the sensitivity and specificity of cytological testing using fluorescent in situ hybridisation probes, however, this technique remains expensive and not in widespread clinical use.^3^ Once diagnosis is established, treatment options for CC are limited. Radical surgical resection requires an extensive, prolonged procedure and less than 7% of patients have disease amenable to surgery. Liver transplantation is being pioneered as a potential option for selected patients with CC, however, many patients who are intensively screened for this potential curative procedure are found to not be eligible and do not complete the rigorous neoadjuvant regimen of chemoradiation. Furthermore, the long term outcomes on survival or quality of life following liver transplantation for CC are unknown.^4^ Studies comparing chemotherapy either alone or in combination for patients with unresectable disease have demonstrated partial disease response rates in the order of 10–30% but only modest effects on overall survival.^5–8^ Trials have shown an improvement in progression-free and overall survival of approximately 3 months in patients receiving combined gemcitabine/platinum-based chemotherapy compared to gemcitabine alone.^9^ These results were corroborated in a Japanese population with similar effects on outcomes.^10^ This combination of chemotherapy is now the accepted standard of care for patients with advanced CC. Phase II randomised controlled trials of monoclonal antibodies to the receptor tyrosine kinases EGFR and VEGFR (known to be overexpressed and functional in CC) have been disappointing despite encouraging early results in pre-clinical studies.^11–13^

Such absence of efficacy in CC, using agents that are well established to be highly beneficial in other gastrointestinal cancer types, including metastatic colorectal cancer, is disappointing. Novel therapeutic avenues, therefore, need to be explored and this requires an improved and detailed understanding of the events leading to the initiation and development of CC, how the tumour is sustained, supported and promoted by its highly desmoplastic stromal environment and what signals might be targeted for new treatments.

## The mechanisms underpinning liver regeneration following injury

**Fig. 1:**
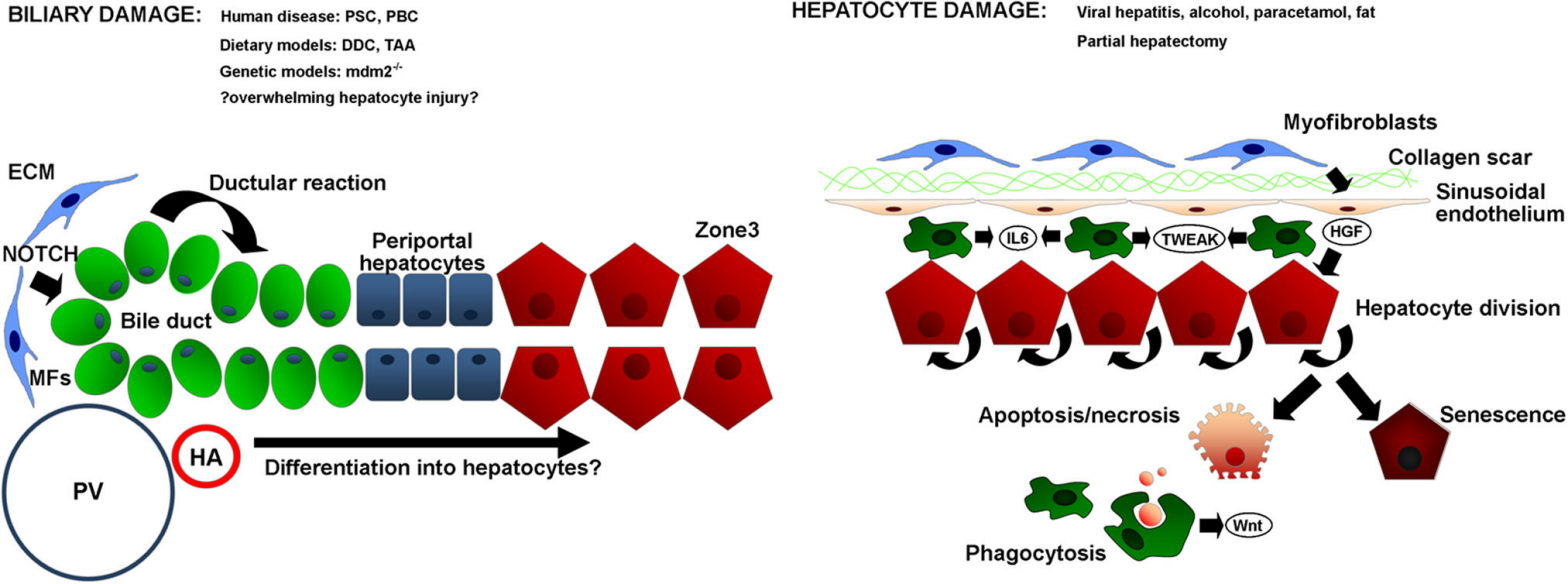
Mechanisms of parenchymal repair in the adult liver after injury. Schematic micrograph showing the contribution made by hepatocytes vs. hepatic progenitor cells (HPCs) to parenchymal repair under conditions of biliary or hepatocyte damage. In response to biliary injury there is mobilisation and proliferation of hepatic progenitor cells within a laminin rich niche via myofibroblast-derived signals including Jagged1 to activate the ‘ductular reaction’. In contrast, hepatocyte targeted injuries including partial hepatectomy stimulate hepatocytes to enter mitosis and restore liver mass. In the absence of additional superimposed injury, this capacity is almost infinite (up to 90% of liver volume can be resected in rat), but becomes overwhelmed following chronic or fulminant damage when hepatocytes either undergo necrosis or become increasingly senescent. It is thought phagocytosis of hepatocyte debris stimulates the HPC response via signals including macrophage-derived Wnt. *PV* portal vein, *HA* hepatic artery, *MF* myofibroblast, *ECM* extracellular matrix

In contrast to organ systems such as skin or blood, homeostatic regeneration of the normal liver is not thought to be dependent upon stem cell-derived epithelial repopulation.^14^ Following prolonged or severe liver injury the cellular mechanisms of regeneration of the liver may change, and much research has focussed on determining the contribution of hepatocyte self-replication vs. expansion of the putative hepatic progenitor cell (HPC) population resident in the bile ductules (Fig. 1). There is consensus that during homeostasis parenchymal turnover is maintained solely through hepatocyte division controlled by a selected number of ‘master regulator’ signals including Wnt/β-catenin and Hippo/Yap.^15, 16^ This spatiotemporal regulation contributes to metabolic zonation and determines hepatocyte function.^15, 17^ More controversial is the evidence for the cellular source of parenchymal repair during liver regeneration after injury. Rodent models including the classical experiment of partial hepatectomy in the rat, demonstrate that liver size is restored through hepatocyte hypertrophy and hyperplasia in the remaining lobes; a highly regulated process dependent on changes in blood flow, the sinusoidal endothelium, immune cells, stellate cells and a host of growth factors and paracrine signals.^18, 19^ This regenerative potential is both highly efficient and of almost infinite capacity.^20, 21^ It also appears that when the ability of hepatocytes to proliferate is inhibited or overwhelmed, the liver maintains the ability to regenerate via mobilisation of a population of HPCs.^22, 23^ However, much debate has surrounded the true significance of this HPC pool. In zebrafish models, where hepatocytes are ablated or prevented from entering the cell cycle, HPCs are activated, proliferate and can restore the hepatocyte mass ensuring survival of the organism.^24^

Recent work has proposed a third regenerative mechanism in the mammalian liver; the transition of hepatocytes into the biliary state or hepatocyte ‘transdifferentiation’.^25, 26^ Hepatocyte labelling studies using chimaeric livers or Cre-based systems have demonstrated the appearance of biliary-exclusive labels including CK7 in hepatocyte-derived cells following injury, especially in the context of bile duct ligation (BDL) or toxin-mediated damage.^25, 27^ This process can be activated by Notch signalling, as occurs in the embryonic liver where Notch ligand supplied by the portal mesenchyme induces differentiation of hepatoblasts into biliary epithelia.^28^ Ectopic expression of Notch 1 in fluorescently labelled adult hepatocytes, using viral delivery of Cre induces co-expression of hepatocyte and biliary markers as well as changes in polarity and morphology so that the cells adopt a more biliary morphology.^26^ The appearance of this intermediate or ‘biphenotypic’ population of cells has been observed in a number of rodent models of injury and it is postulated that this occurs in human disease as part of an in vivo cellular reprogramming as a response to biliary-specific damage.^26^

## Defining the cell of origin in CC

**Fig. 2:**
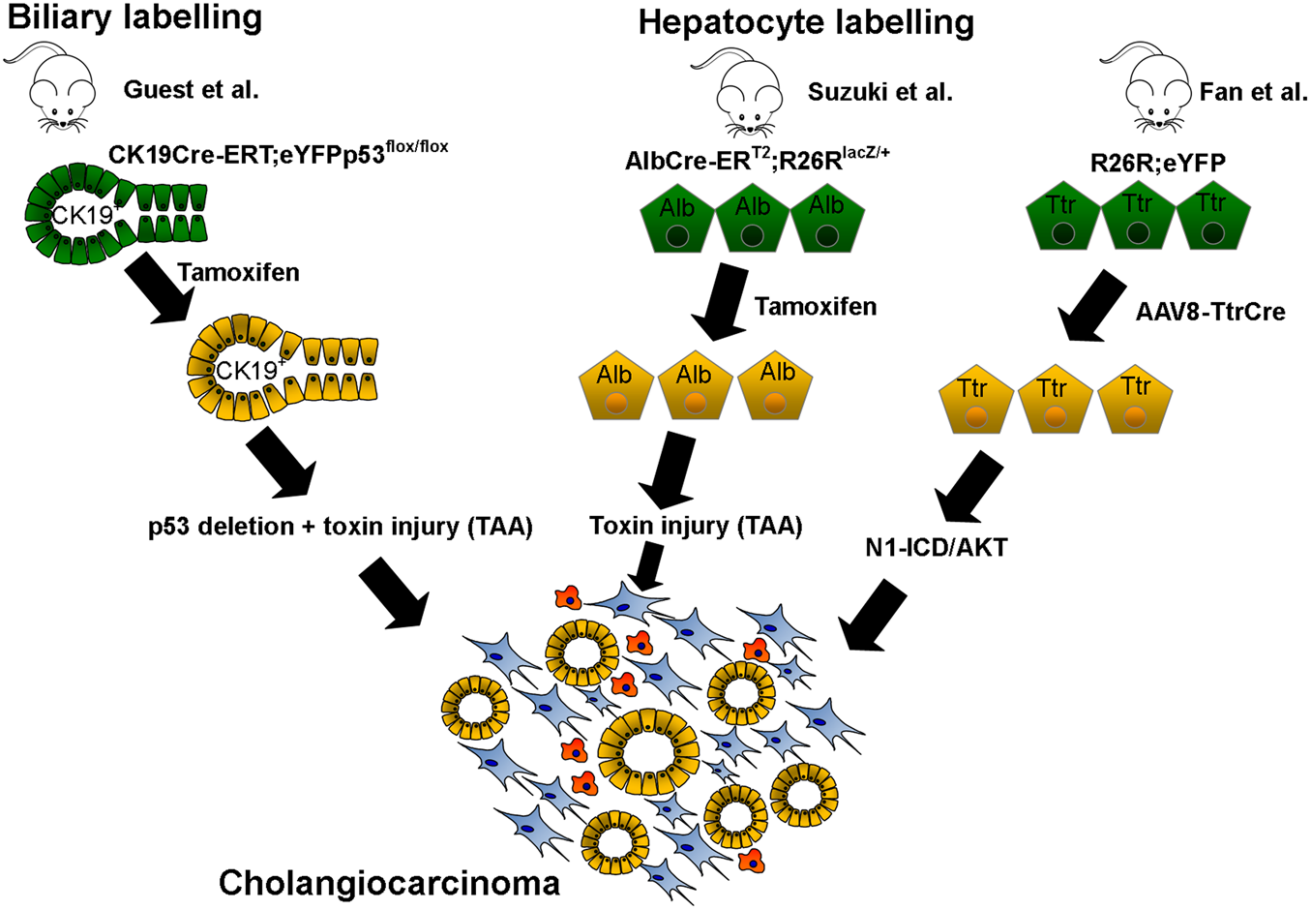
The cell of origin of cholangiocarcinoma. Schematic micrograph of lineage tracing experiments in mouse demonstrating the contribution of cytokeratin-19-positive ductular cells and albumin or transthyretin-positive hepatocytes to the formation of tumours exhibiting biliary differentiation. Cholangiocarcinoma can arise from either cholangiocytes, immature ductules or through transdifferentiation of hepatocytes

Similar to the cellular response to benign liver injury and regeneration, it is now clear that such plasticity of the two epithelial liver cell types is also exhibited in response to oncogenic stimuli (Fig. 2). The historical presumption that CC arises from bile ducts was based on pathological observations of tumours arising from or adjacent to ductal epithelia or invading into ductal lumina in addition to immunoreactivity of tumours for cholangiocyte-specific proteins including CK7 and CK19 (ref. 29). Liver tumours are described which display features of both CC and hepatocellular carcinoma; so-called combined hepatocellular cholangiocarcinoma, with a histopathological subset classified as cholangiocellular carcinoma (CLC) exhibiting ductular reaction and cord-like structures resemblant of the Canals of Hering and are both postulated to arise from progenitor cells.^30^ Overall these represent a small minority of tumours with biliary differentiation and the association of CC with chronic biliary inflammation in particular chronic liver fluke infection and primary sclerosing cholangitis (PSC), has added weight to the theory that CC arises from cholangiocytes.^31, 32^ However, this assumption has been challenged following work applying lineage tracing systems to models of CC. Interpreting these experiments in mouse that aim to identify the CC cell of origin has been controversial, in part due to the widely used Albumin-Cre system. Albumin expression in the perinatal and juvenile liver mirrors the organ’s transition from a haematopoietic to hepatic function and, therefore, constitutive albumin Cre systems result in ductular labelling in the adult.^33^ This effect has been borne out in a model where *kRas* mutation and *Pten* deletion are used to induce CC under control of the Albumin promoter. CC is observed in mice receiving Cre induction shortly after birth (P10), but not at later post-natal time points (P42) (ref. 34).

In contrast, labelling in the adult using an inducible biliary-specific cytokeratin 19 driven Cre system does result in CC arising from cholangiocytes, when p53 is deleted from cholangiocytes and then mice are subjected to chronic toxin-mediated damage.^35^ Histological heterogeneity displayed by CC arising from different locations along the bile duct supports the findings from immunohistochemical and gene expression data that subsets of CC exist; likely arising from cholangiocytes of differing differentiation states or maturity. For example, perihilar and some intrahepatic CCs exhibit expression signatures mirroring those of mature, mucin-producing cholangiocytes of the large ducts, which share embryological origins with extrahepatic and pancreatic ductal cells. In contrast other CCs exhibit profiles aligned with those of the HPCs of the terminal ductules (CLCs).^36^

Until recent times the hepatocyte had not been evaluated as a potential cell of origin for CC, however, given the common embryological origin of the two cell types and the occurrence of perivenular CC in the context of chronic hepatocyte injury, e.g., hepatitis C virus infection, the hypothesis that mature hepatocytes could be a cell of origin of CC would appear conceptually plausible. Indeed there is now good evidence that hepatocyte conversion into CC is possible when exposed to favourable stimuli, and the key driver of this process appears to be Notch signalling.^37, 38^ Again the use of heritable labelling with inducible Cre-loxP technology was employed to ‘tattoo’ cells expressing the albumin locus (at 8 weeks post-natally to ensure exclusive hepatocyte tracing) (Alb-Cre-ER^T2^;R26R^lacZ/+^) or the CK19 locus (CK19-Cre-ER^T2^;R26R^lacZ/+^). After carcinogen exposure using thioacetamide, lacZ-positive tumours with features of biliary differentiation (i.e., CC) were observed only in the Albumin and not the CK19 labelled animals, and arose from centrilobular hepatocytes. To help explain this interesting finding, it was proposed that the P450 cytochrome enzymes required to produce the carcinogenic metabolite from thioacetamide are located in this region. To confirm the requirement of Notch signal in this conversion, the authors of the study used a construct to either overexpress the intracellular fragment of Notch1 (N1-ICD) (Alb-CreER^T2^;R26R^Notch/+^) or delete the principal effector of canonical Notch, Hes1 (*Alb-CreER* ^*T2*^;*Hes1* ^*fl/fl*^). They observed an increase in the number of neoplastic ductular nodules in response to Notch1 over-expression, whereas nodules did not form in animals with deletion of Hes1 in Albumin-expressing cells.^38^ These findings have been corroborated by an alternative fate-tracing study using plasmid delivery of N1-ICD/AKT into livers of R26ReYFP mice where hepatocytes were labelled using transthyretin-driven Cre (delivered using adeno-associated virus). The resulting tumours expressed eYFP demonstrating they arose from hepatocytes. One criticism of this paper has been the selection of biliary markers used to demonstrate biliary differentiation of tumours, in particular Sox9 and CK8, which are widely regarded to be also expressed by hepatocytes.^37^ CC can arise from cells at any point along the biliary tree; from the large extrahepatic ducts to the terminal small ductules within the liver.

The genetic tracing evidence supporting further intra- and inter-tumoural diversity in the cellular origin of CC arising from the liver parenchyma has deep implications for therapy and adds further complexity to what is already known to be a highly heterogeneous malignancy. Gene expression analysis can distinguish between tumours arising from different cells of origins as demonstrated by Holczbauer and colleagues who virally transformed cells at different stages along the hepatic differentiation lineage (mouse HPCs, lineage-committed hepatoblasts or adult differentiated hepatocytes) and demonstrated genetic and phenotypic heterogeneity in the resulting tumours.^39^ The implication that differing cells of origin in tumours between patients and potentially within one individual suggests huge divergence of somatic mutation profiles, differences in epigenetic landscapes, activation of signalling pathways and ultimately may well explain the large variability in patient response to therapy. Therefore, in the future, adopting a molecular diagnostic approach to CC may pave the way for personalised medicine where the therapeutic agents offered could have greater efficacy in that particular sub-group of CC disease.

## Drivers of the cell identity in liver development and regeneration drive CC: Notch, Yap and Wnt

**Fig. 3:**
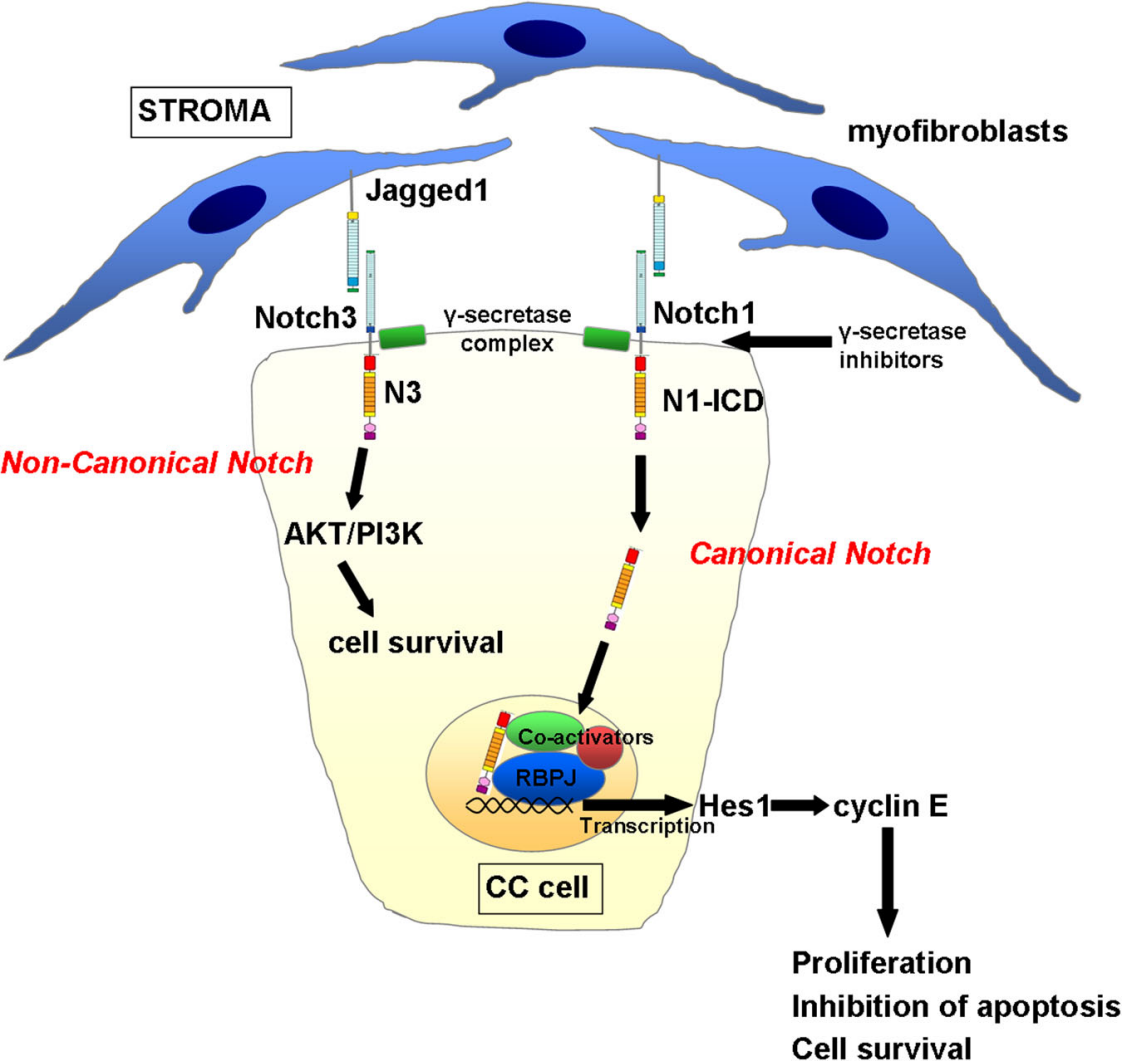
Notch is over-activated in CC and drives tumour cell survival. Schematic micrograph of the provision of Notch ligand (Jagged1) by myofibroblasts in the CC stroma; triggering Notch activity in adjacent CC cells. Notch1 and the atypical Notch3 receptor are over-expressed in human and rodent models of CC, and both appear to drive CC proliferation. Notch1 acts via the canonical pathway, releasing Notch1 intracellular domain (N1-ICD), which translocates to the nucleus, drives transcription of classical Notch effectors including Hes1, which then promotes proliferation through expression of genes including Cyclin E. Notch3 appears to stimulate activity through the AKT/PI3K cell survival pathway independent of the effector of canonical Notch, RBPJκ. A further level at which the pathway can be therapeutically targeted is the point of receptor cleavage in the membrane. Inhibitors of the enzyme complex gamma-secretase have demonstrated anti-tumoural activity in cell culture systems, xenografts and toxin driven models in rat

The role of Notch as a crucial determinant of tumour cell phenotype is consistent with its established role in liver development, as previously emphasised, where hepatoblast differentiation is dependent upon the cell’s location relative to the portal vein mesenchyme and hence exposure to the Notch ligand JAG1, as well as other signals including TGFβ.^40^ Evidence from mouse studies suggest that Notch activation can be a key driver of CC development, regardless of the cell of origin in which the initiating mutational events occur (Fig. 3). Canonical Notch signalling in mammals is characterised by a cell-to-cell signal in which membrane tethered ligand (Jagged (*JAG*)1 and 2 and Delta-like ligand (*Dll*)1, 3 and 4) can interact with any one of four receptors (*NOTCH1*, *NOTCH2*, *NOTCH3* and *NOTCH4*). This triggers a series of enzymatic cleavage events within the membrane of the signal-receiving cell, mediated principally by a complex known as gamma-secretase. This proteolysis releases a truncated form of the receptor, the intracellular domain (N-ICD), which is then able to translocate to the nucleus where it acts to liberate its downstream target genes from a state of active transcriptional repression. N-ICD forms a complex with its DNA-binding partner Recombinant Signal Binding Protein for Immunoglobulin Kappa J (*RBPJκ*), which triggers displacement of co-repressors with the co-activators *MAML* and *SKIP*, allowing transcription of the Notch target genes, which include the Hairy Enhancer of Split (Hes) family.^41, 42^

Mutations of the Notch family are known to be associated with aberrant liver development. For example, loss of function mutations of *JAG1* and less frequently *NOTCH2* result in Alagille’s syndrome; an autosomal dominant disorder resulting in a failure of development of the intrahepatic bile ducts leading to cholestasis and jaundice.^43^ Transforming mutations of Notch are not, however, observed in CC.^44–46^ Rather it appears to be activation of wild-type signalling that leads to ductular proliferation and/or a change of lineage commitment in hepatocytes, as has been exemplified by studies over-expressing wild type N1-ICD in hepatocytes to produce CC.^37^ It should, however, be stated that the precise role of Notch in liver cancer development is complex. Over-expression of N1-ICD under albumin and αFP promoters in developing mouse liver results in HCC with 100% penetrance without the appearance of CC.^47^ Furthermore, studies using blocking antibodies against Notch1 results in a reduction in HCC but an increase in CC, whereas blocking Notch2 or Jag1 reduced CC burden.^48^ Studies of other oncogenes including *MYC* and *RAS*, have demonstrated that precise levels of expression are critical to outcome.^49^ It is likely that transgenic models using N1-ICD overexpression result in supra-physiological levels of Notch activity that are unlikely to occur naturally in response to endogenous ligand-driven receptor activation. A small number of studies have, therefore, sought to characterise the role of the four Notch receptors in human CC. These have shown Notch1, 2 and 3 to be over-expressed human CC compared to nearby healthy or benign diseased liver and that development of CC can be inhibited in vitro and in vivo with small molecule inhibitors of the gamma-secretase complex (GSIs).^50, 51^ Furthermore, there is evidence that genetic deletion of the atypical receptor Notch3 can inhibit CC development and progression in mouse and that Notch3 is able to promote tumour cell survival through activation of the PI3K/AKT pathway.^52^ Notch3 differs structurally from the more intensively studied Notch1 and Notch2, in that it exhibits a particularly short transactivation domain; thought to account for its weaker transcriptional activity, as well as lacking some of the extracellular EGFR repeats of Notch1 and 2 (refs 53, 54). The precise route by which it is able to trigger PI3K/AKT signalling remains unclear, however in vitro data suggest that this is not dependent on the effector of classical Notch, RBPJκ. These new data, therefore, offer hope for a novel therapeutic approach, having the potential therapeutic advantage that specific Notch3 blockade might bypass toxicities associated with Notch1 and gamma secretase inhibition.^55^

Other signals of cell identity implicated in liver tumorigenesis include the mammalian Hippo-Salvador pathway. This pathway is tonically active in hepatocytes via the kinases Mst1 and Mst2, acting to control hepatocyte proliferation and act as tumour suppressors via phosphorylation and, therefore, suppression of the downstream target Yes-associated protein (Yap). Genetic deletion of *Mst1* and *Mst2* results in massive liver overgrowth and eventually HCC development.^56^ Disruption of the pathway through liver-specific knock out of WW45, a homolog of *Drosophila* Salvador, similarly results in liver enlargement accompanied by an HPC response, mediated through phosphorylation and hyperactivation of the downstream effector, Yap. Eventually these animals develop tumours exhibiting mixed HCC/CC characteristics; thought to derive from the expanded HPC pool.^57^ Furthermore, transposon-delivery of YAP and constitutively activated AKT (myr-AKT) results in the development of CC in mouse when coupled with BDL, with tumours exhibiting biliary but not hepatocellular markers, confirming YAP to be oncogenic in CC.^58^ YAP has been shown to be activated in human disease and is thought to promote CC growth via interaction with TEAD transcription factors to stimulate proliferation, inhibit apoptosis and promote angiogenesis.^59^

Wnt is a central regulator in many epithelial systems including the skin, intestine and gut where canonical, β-catenin-dependent WNT signalling drives stem cell and differentiated cell proliferation by inducing the transcription of a number of target genes including MYC and CCND2 (ref. 60). Mutations in the core canonical Wnt pathway have been shown to drive several gastrointestinal and other cancers, most notably colorectal cancer where mutations in APC and less so in beta-catenin are known oncogenes.^61^ Recent exome studies have failed to identify these canonical mutations in CC, with no APC, β-catenin or Axin mutations found.^62^ In a subset of liver fluke-associated intrahepatic CCs, however, mutations in the E3 ligase, RNF43 ((Ring Finger Protein 43, a negative regulator of Wnt signalling) were found in 7.4% of CC from patients with liver fluke and 3.5% of non-liver fluke-associated CC. RNF43 normally functions to turn over the Frizzled (Fzd) receptor following ligand binding by ubiquitinating Fzd and targeting it for degradation. R-spondin and Lgr5 negatively regulate RNF43 (and its homologue ZNRF3) thereby potentiating Wnt signalling through the canonical pathway. Loss of function mutations in RNF43 that, therefore, promote Wnt signalling through failing to downregulate the receptor following stimulation thus allow the Fzd receptor to be hyperactivated.^63^ Despite the lack of core mutations, it remains that there is a high level of canonical Wnt activity in sporadic CC, typified by high levels of nuclear b-catenin of CC cells in ~ 76% of cases. Recent work has demonstrated that activation of the Wnt pathway is achieved through the influx of inflammatory macrophages in the tumour stroma which are able to secrete Wnt ligands, particularly WNT7B, which acts upon the epithelial component of the tumour to drive proliferation and tumour growth.^64, 65^ Of therapeutic importance, pharmacological inhibition of Wnt signalling either at the point of transcription by preventing binding to CtBP or through inhibition of ligand secretion through inhibition of the MBOAT-family member Porcupine, reduces both the size frequency of tumours in rat and mouse CC models by disrupting the balance of proliferation and apoptosis.^64^ Further evidence of disruption of the canonical Wnt pathway has been through demonstration of methylation of Wnt pathway regulators, particularly the secreted frizzled-related protein family (SFRPs), which act as soluble negative modulators of Wnt signalling.^66^ Epigenetic silencing of SFRP2 is proposed to act to stabilise beta-catenin as competition at the Frizzled receptor is reduced or lost, although in this study no correlation was demonstrated between SFRP2 and beta-catenin positivity (nuclear or cytoplasmic) in human tissue.

As well as proliferation, the canonical Wnt pathway is also capable of regulating the expression of transcribed-ultraconserved regions (T-UCRs), long non-coding RNAs, which are involved in various cancers. Recently, uc.158, a T-UCR downstream of the Wnt/β-catenin pathway, has been found to be increased in TAA-induced rat and human CC but not in normal biliary epithelium. Furthermore, the TAA-induced rats treated with Wnt inhibitors had reduced levels of uc.158, suggesting the effects of Wnt signalling in CC might not simply be driving proliferation, but also feed into a number of secondary pathways through T-UCR regulation.^67^

## Inflammation, fibrosis and repair: the tumour stroma

In their seminal paper, Hanahan and Weinberg described six adaptations a cell may acquire in order to escape the homeostatic processes, which enable it to function as part of an organ.^68^ These characteristics; self-sufficiency in growth signals and the ability to evade growth supressing signals, ability to proliferate indefinitely and escape death signals, angiogenesis and metastatic potential, describe a cell that has acquired the ability to function beyond its role as part of an organ system, and is more like its own organism. It is becoming clear that as well as these changes, a seventh hallmark of cancer is its ability to remodel the inflammatory and wound healing response to organ injury to promote growth and evade destruction by the adaptive immune system. As previously discussed, CC commonly arises on a background of cholestatic injuries, such as PSC and liver fluke infection. These injuries, although diverse in origin, share a phenotype of cholangiocyte damage, a pro-inflammatory immune environment, deposition of extracellular matrix proteins and consequent peri-ductular scar formation. A defining feature of CC is its prominent desmoplastic stroma consisting of α-smooth muscle actin (α-SMA)-positive cancer-associated fibroblasts and numerous immune cell types including tumour-associated macrophages (TAMs), neutrophils and vascular endothelial cells.^69^ The presence of a well-developed fibrous stroma negatively correlates with CC survival and more recent studies have established the importance of the tumour stroma in maintaining tumorigenic identity of CC, presenting the possibility of therapeutically targeting the stroma.^70^ In this section, we will discuss the cellular crosstalk between these stromal cells and CC cells, and the implications of these interactions with respect to the pathogenesis of CC and potential therapeutic targeting of these pathways.

CAFs are activated α-SMA^+^ mesenchymal cells that can be derived from activated hepatic stellate cells, the pericyte of the liver and potentially from other cells sources.^71–73^ The prevalence of α-SMA^+^ CAFs in tumours correlates with poor clinical outcomes in CC patients.^72, 74^ Investigation of interactions between CAFs and tumour cells show that CAFs support many of the malignant features of CC including proliferation, migration and evasion of apoptosis via secreted factors.^73^ An example of this is paracrine Hedgehog (HH) signalling. Hedgehog is an established regulator of cell fate in the mammalian liver and in regeneration the obligate intermediate of Hedgehog signalling, Smoothened (SMO) acts to control accumulation of activated myofibroblasts and progenitor cells following injury, integrating other pathways including Wnt and TGFβ for co-ordination of the fibrotic and regenerative responses.^75^ Furthermore, Hedgehog appears to also regulate Yap1 in stellate cells, activating genes to trigger the transition to activated myofibroblasts and promote fibrosis.^76^ Human CC cells appear to both express and be responsive to the Hedgehog ligand Sonic Hedgehog (Shh), and Shh pathway inhibition reduces CC growth, invasion and migration and promotes CC cell apoptosis.^77^ Secretion of PDGF-BB signalling by CAFs further reinforces CC survival by augmenting Hh signalling in CC cells via upregulation of SMO resulting in resistance to TRAIL-mediated apoptosis.^78^

With regard to in vitro CAF data, co-culture of CC cells with mesenchymal cells induces secretion of several cytokines/chemokines including EGF, IGF-1, PDFG-BB and TGF-β1 in CC cells. The co-culture experiments suggest a two-way trophic effect as this induces EGF and TGF-β1 secretion by LX-2 stellate-like cells.^79^ HB-EGF produced by CAFs or CC cells themselves induces proliferation and EMT of CC cells and is reinforced by CC-derived TGF-β1.^80, 81^ PDGF-D secreted by neoplastic biliary epithelial cells also increases motility of fibroblasts, which may be important for the development of the tumour stroma.^82^ Although CAF-CC interactions promote CC survival, activation of hepatic stellate cells into activated myofibroblasts is accompanied by the upregulation of the pro-apoptotic protein, Bak. This property has been used to specifically induce apoptosis in CAFs in an orthotopic rat model of CC, using the Bcl-inhibitor drug navitoclax, which limited tumour stroma formation, reduced tumour burden and significantly improved animal survival providing promising evidence that therapies targeting CAF viability may limit CC growth.^83^

In addition to EGF/EGFR interactions, the malignant properties of CC cells are enhanced by the SDF/CXCR4 pathway and IL-1β -CXCL5, which also interface with the infiltrating immune compartment of the tumour stroma. Stromal SDF-1 signals to CXCR4^+^ CC cells to increase their migratory potential and resistance to apoptosis via p-AKT and p-ERK signals.^84, 85^ This interaction is enhanced by mononuclear cell-derived TNF, which modulates CXCR4-dependent migration.^85^ Autocrine CXCL5 from CC cells also activates AKT and ERK pathways, increases CC motility and its secretion is enhanced by CAF-secreted IL-1β.^86^ CXCL5 is a potent neutrophil chemoattractant in CC and its expression is positively correlated with the numbers of α-SMA^+^ CAFs and CD66b^+^ neutrophil infiltration.^86^ Although the role of tumour-associated neutrophils (TANs) has not been extensively studied, neutrophils recruited to the CC niche increase tumour growth and metastatic potential, and correlate with reduced post-operative survival and increased post-operative recurrence of disease, suggesting that targeting the trafficking of TANs to the tumour niche might provide a viable therapeutic intervention following tumour resection in CC.^86, 87^

The major compartment of infiltrating immune cells in CC are TAMs, which correlate with poor survival, increased tumour recurrence and metastasis.^88, 89^ TAMs are most likely recruited from a subset of circulating CD14^+^/CD16^+^ monocytes, which is elevated in CC patients,^89^ to the tumour microenvironment where they give rise to TAMs, which are actively patterned towards an ‘M2’-like CD163^+^ phenotype^88, 90^ and express proteins that promote tumour growth and progression including matrix metalloproteases,^88, 89^ Wnt ligands (discussed above)^64, 65^and cytokines such as TNF, IL-4, IL-6, IL-10 and TGFβ, which promote tumour progression by inducing EMT.^88, 91, 92^ The effect of deleting TAMs has been shown by Boulter et al.^64^ in the rat TAA model of CC, here the deletion of TAMs by liposomal chlodronate significantly reduced tumour size, induced tumour cell apoptosis and reduced Wnt signalling.

## How novel data will help overcome challenges in the field of CC

There are several clinical factors that lead to the poor prognosis of CC: (1) A significant factor is the delay in diagnosis and patients often present at a point where curative surgery is not possible. Clearly, the identification of secreted biomarkers (either made by the tumour, the stroma or the immune system in response to the CC) would have great utility in the potential early diagnosis of CC. This would be particularly useful where there is already an inflammatory process affecting the bile ducts such as PSC or fluke infection that can pre-dispose to CC. (2) the lack of effective therapy to unresectable CC. Here recent work detailing the heterogeneity of CC and the signals and cell processes that drive CC could be particularly useful. Molecular analysis of the tumour can potentially lead to treatment stratification based upon the main cancer drivers upregulated in that particular tumour. Likewise, the recognition that there is heterogeneity in the potential cell of origin helps us to unpick future targets with potentially greater specificity. This is a timely approach with the growing recognition and emphasis placed upon personalised medicine. In practical terms this could be from cancer brushings and biopsies either analysed directly for their molecular profiles or as research protocols being grown as organoids to test sensitivity to chemotherapies and other agents.

## Summary

Recent rapid process has been made in understanding the signals driving CC. These have often been the same signals that have been described in bile duct development and regeneration following injury such as Wnt and Notch. The clarification of the potential for hepatocytes and cholangiocytes to act as the cell of origin for CC has opened the pathway to understand further the drivers for CC. There has been an increasing recognition that the tumour stroma is an important component of the CC, providing trophic signals to the epithelial cancer and potentially providing resistance to chemotherapy. Understanding the signals and mechanisms that drive CC will help the development of novel therapies and importantly the development of personalised therapy for CC. The next few years are likely to see continued progress into the diagnosis and treatment of CC, information that is greatly needed for this devastating cancer.
